# Cemented fixed-bearing PFC total knee arthroplasty: survival and failure analysis at 12–17 years

**DOI:** 10.1007/s10195-011-0142-2

**Published:** 2011-06-23

**Authors:** A. Bistolfi, G. Massazza, F. Rosso, D. Deledda, V. Gaito, F. Lagalla, C. Olivero, M. Crova

**Affiliations:** 1 Department of Orthopedics and Traumatology, CTO/M. Adelaide Hospital, Turin, Italy; 2 Department of Orthopedics and Traumatology, University of the Studies of Turin, Turin, Italy; 3 c/o AO CTO/M. Adelaide, Via Zuretti 29, 10126 Turin, Italy

**Keywords:** Total knee arthroplasty, Long-term survival, Failure analysis

## Abstract

**Background:**

Total knee arthroplasty (TKA) is the appropriate treatment for degenerative pathology of the knee. Implant surveillance is mandatory to improve clinical results. We present the long-term results of a series of consecutive TKA Press Fit Condylar (J&J), cemented fixed bearing with selective patellar resurfacing in nonselected patients.

**Materials and methods:**

In this prospective case series, 223 TKA were clinically and radiographically evaluated using the Hospital for Special Surgery (HSS) knee score and the Knee Society Roentgenographic Evaluation and Scoring System.

**Results:**

There were 197 patients, with an average age of 68.4 years [95% confidence interval (CI) 52.7–84.1 years]; 49 arthroplasties were implanted in men (21.1%) and 184 (78.9%) in women. The average follow-up was approximately 13.5 years (162.1 months; 95% CI 132.3–191.9), and it was possible to evaluate 179 implants (76.8% of the implanted prosthesis) in 176 patients. The average HSS score increased from 61.5 (95% CI 60.4–62.7) to 89.4 (95% CI 87.7–.93.5) points. The cumulative average survival rate at 15 years (the endpoint being failure with revision) was 90.6%  ± 2% standard deviation. Resurfacing the patella did not make a difference in terms of implant survival. Progressive radiolucent lines were observed around 20 implants (14.3%); all were revised.

**Conclusions:**

The PFC system is an excellent prosthetic solution. Early clinical complications, mechanical axis and patellar resurfacing do not correlate with implant failure, whereas progressive radiolucent lines do.

## Introduction

Total knee arthroplasty (TKA) is the appropriate treatment for degenerative pathology of the knee. Among many existing implants, the Press Fit Condylar (PFC) total knee prosthesis (Johnson & Johnson, Raynham, MA, USA) was introduced in the 1980s to obtain a long-term, strong interface between prosthesis and bone. Despite the subsequent introduction of rotating and uncemented implants [1–3], the cemented models with a fixed bearing still represent the gold standard, with good results and survival reported beyond 10 years [4, 5]. Nevertheless, the primary reason for TKA failure is still not clear, and several problems such as malalignment, wear, loosening, infections, unexplainable persistent pain, etc. have been observed. In addition, it is still a subject of debate whether or not patella resurfacing should be performed. Therefore, long-term prospective surveillance of the implants and analysis of the causes of failure are still of interest to knee surgeons. In this study, we analysed a series of cemented TKA, with a fixed bearing and selective patellar resurfacing, in nonselected patients operated randomly by different surgeons of the same orthopaedic division. Compared with the majority of studies, in which a series of prostheses implanted by a single surgeon is usually reported, participation of several independent surgeons in this study provided a greater possibility for data reproducibility.

## Materials and methods

This was a prospective case series of 233 TKA performed from 1993 to 1998 by various surgeons, using random rotation, in 197 patients (36 bilateral), with PFC fixed-bearing prostheses. Indications to surgery were: 170 (72.9%) patients with knee arthritis with varus–valgus axial deviation <10°, 27 (11.6%) with rheumatoid arthritis, 16 (6.9%) with knee arthritis associated with varus–valgus axial deviation >10°, 11 (4.6%) with arthritis following tibial osteotomy, eight (3.6%) with consequences of tibial plateau fractures and one (0.4%) with failure of unicompartmental prosthesis.

### Surgical technique and protocols

All surgeries were conducted with the patient under general or spinal anesthesia using the same technique and the medial parapatellar approach and capsulotomy, with ischaemic limb (pneumatic tourniquet at 300 mmHg). After removing osteophytes and cruciate ligaments and after a partial release, the distal femoral cut was performed using an intramedullary guide. The cut, with a variation from 0° to 9°, was based on the angle measured in preoperative planning [6]. Thereafter, the ligamentous balance was completed, and the tibial cut was performed. The definitive implant was cemented after choosing a polyethylene liner of appropriate size and after evaluating the range of motion and ligamentous balance. The technique for selective resurfacing was chosen for treating the patella [7]: patellar substitution only in cases of severe cartilage damage, serious deformity and wrong- racking; patellar conservation in the remaining cases. The PFC system offers the choice of either posterior cruciate ligament substitution or retention [8, 9]. All implants were posterior cruciate ligament substituting. The prosthesis also allows the use of a femoral or tibial stem when an augmented stability is required. All patients received an antibiotic prophylaxis approximately 1 h before surgery with a single dose of a single antibiotic and an antithromboembolic with low molecular weight heparin for 30 days.

### Clinical evaluation

The Hospital for Special Surgery (HSS) knee score questionnaire was used for clinical evaluation [10]. Patients underwent clinical evaluation at the outpatient departments dedicated to prosthetic surgery. Evaluation was conducted prior to and after implantation at 3 and 6 months and then annually. In addition, personal identification data, concomitant pathologies and complications that occurred after implantation were recorded. When poor conditions of general health or a long distance from the place of residence made the transfer of clinical and radiographic control at our hospital not possible, patients responded to a telephonic questionnaire. This is in agreement with the literature that supports the quality of data obtained using the telephonic method [11].

### Radiographic evaluation

The Knee Society Roentgenographic Evaluation System [12] was used with the objective of obtaining a standardised radiographic evaluation. All patients had an immediate postoperative radiographic control in the operating room, followed by a definitive radiographic evaluation with weight bearing in the anteroposterior (AP) and laterolateral (LL) projections at the same time as the clinical evaluations. X-rays were studied to observe implant position, alignment angle (α, β, γ and δ), eventual signs of periprosthetic fractures, mobilisation or loosening and the presence of osteolytic areas. Radiolucent lines were recorded (defining radiolucent lines as the distance of the bone–prosthesis interface >2 mm). These lines were subdivided into not progressive, which indicates lack of mobilisation; and progressive, which is a sign of probable implant loosening. The analysis was performed on digitised X-rays present in our hospital’s database and on the films produced by the patient, for X-rays taken in other hospitals.

### Statistical analysis and failure evaluation

All data were collected in perspective through a dedicated computer programme created to manage prosthetic follow-up. This programme also allowed collection of radiographic parameters and successive statistic analysis. Clinical and radiographic data were analysed using means, standard deviations (SD) and confidence intervals (CI). Statistical significance for all data was set at *P* < 0.05. The Kaplan–Meier method with two different endpoints was performed for survivorship analysis: The first endpoint was prosthetic revision to provide a total evaluation of implant survival. An HSS score of 60 points was chosen as the second endpoint to give importance to patient satisfaction. Differences in cumulative survivorship in the patellar replacement group and the group with no patellar replacement were evaluated using the log-rank test.

## Results

Follow-up concluded in December 2009. From the initial 233 implants, the right knee was replaced in 116 cases (49.8%) and the left knee in 117 (50.2%) cases . Forty-nine arthroplasties were implanted in men (21.1%) and 184 (78.9%) in women. The average age at surgery was 68.4 (95% CI 52.7–84.1) years. A femoral or tibial stem was used in five cases (2.1%); the patella was replaced in 144 cases (72.9%). During the follow-up period, 15 patients died without signs or symptoms of failure. In addition, 37 patients did not match the follow-up protocol for almost 2 years and were therefore considered to be lost. Finally, 179 implants (76.8% of implanted prosthesis) in 176 patients were evaluated (107 with patellar resurfaced and 72 with patella not resurfaced). The telephonic evaluation was applied in seven cases. Average follow-up was calculated with respect to the last clinical control (including deceased and lost patients, with shorter follow-up) and was equal to approximately 13.5 years (162.1 months; 95% CI 132.3–191.9 months).

### Clinical results

All HSS score items of the 176 patients had a statistically significant improvement from preoperative to postoperative analysis (*P* < 0.05). The total average score increased from 61.5 (95% CI 60.4–62.7) to 89.4 (95% CI 87.7–93.5) points. Pain at rest improved from 4.4 (severe; 95% CI 4.1–4.8) to 12.4 (absent; 95% CI 11.9–12.9), pain marching from 8.4 (95% CI 7.9–8.8) to 13.4 (95% CI 12.9–13.8) points, functionality from 6.5 (95% CI 6.2–6.9) to 9.7 (95% CI 9.4–10.1) points; range of motion increased from 86.5° (95% CI 84.8°–88.2°) before surgery to 108° (95% CI 106.1°–109.9°). Patellar resurfaced and unresurfaced TKAs were compared, and no statistically significant difference (*P* > 0.05) was observed between the two groups for all HSS items (Table 1).

**Table 1:** Comparison between patellar resurfaced and unresurfaced total knee arthroplasty (*P* value > 0.05 for all items)

	Patella resurfaced	Patella not resurfaced	*P* value
Total HSS	85.9 (95% CI 83.8–88.1)	81.7 (95% CI 78.6–84.9)	0.1053
Pain	24.2 (95% CI 22.6–25.9)	26.7 (95% CI 25.7–27.8)	0.0636
ROM	106.3° (95% CI 102.7°–110.1°)	109.1 (95% CI 106.4°–111.7°)	0.0647
Functionality	17.8 (95% CI 16.8–18.8)	16.8 (95% CI 15.6–18.1)	0.5911
Quadriceps force	9.4 (95% CI 9.2–9.6)	9.1 (95% CI 8.7–9.4)	0.3646
Flexion deformity	9.9 (95% CI 9.8–9.9)	9.8 (95% CI 9.6–9.9)	0.3699
Instability	9.5 (95% CI 9.3–9.7)	9.4 (95% CI 9.1–9.6)	0.0699

*CI*confidence interval

### Complications

A complication after surgery occurred in 36 cases (20.2%): 18 (10.1%) incomplete cicatrisation of the surgical wound, in absence of signs of infection, treated with a superficial revision; nine (5.0%) postoperative haematoma, redness, swelling and temperature, treated with an immediate articular washing and polyethylene exchange; two (1.1%) postoperative periprosthetic incomplete tibial fractures, treated with immobilisation followed by complete final healing; four (2.3%) periarticular calcifications and three (1.7%) peroneal nerve palsy, which were not treated.

### Radiographic results

X-rays of 140 patients (78.2% of the patients who underwent clinical evaluation) were analysed. Radiolucent lines were found in 71 cases (50.7%), but progression of the radiolucent space (index of mobilisation) was observed only in 20 cases (14.3%). These 20 cases underwent a revised implant. A significant correlation between progressive radiolucent lines and failure (revision) was found. Distribution of the radiolucent lines in the tibial and femoral areas is reported in Table 2. The angles described by Ewald [12] were measured: the average α angle was 94.4°, the β angle 89.1°, the γ angle 3.6° and the δ angle 88.6° (95% CI are reported in Table 3). Comparison was made between alignment of implants that were revised for failure and nonrevised implants: no statistically significant differences were found (Table 3).

**Table 2:** Radiolucent line distribution in the different zones of the anteroposterior (AP) and laterolateral (LL) view of the tibia, and of the LL view of the femur, according to the Knee Society Roentgenographic Evaluation System [11]

	Zone	Number of radiolucent lines
Tibia AP	1	40
	4	37
	6	17
Tibia LL	1	34
	2	14
	3	14
Femur (LL)	1	40
	2	6
	3	2
	6	2

**Table 3:** Angles measured on the medial side with respect to the anteroposterior diaphyseal axis for the femoral (α) and tibial (β) components; posterior angles between the axis of the implant and the femoral (γ) and tibial (δ) diaphyseal axis according to the Knee Society Roentgenographic Evaluation System [11]

	α angle	β angle	γ angle	δ angle
All implants	94.4° (95% CI 93.9°–94.8°)	89.1° (95% CI 88.7°–89.5°)	3.6° (IC 95%:3.6°–3.9°)	88.6° (95% CI 88.2°–88.9°)
Revisioned implants	93.5° (95% CI 91.5°–95.5°)	87.9° (95% CI 86.2°–89.6°)	3.7° (IC 95%:2.6°–4.8°)	89.9° (95% CI 87.7°–90.1°)
Nonrevisioned implants	95.5° (95% CI 94.0°–95.1°)	89.3° (95% CI 88.8°–89.8°)	3.6° (IC 95%:3.1°–4.1°)	88.8° (95% CI 88.4°–89.4°)

### Failures and survivorship analysis

**Fig. 1:**
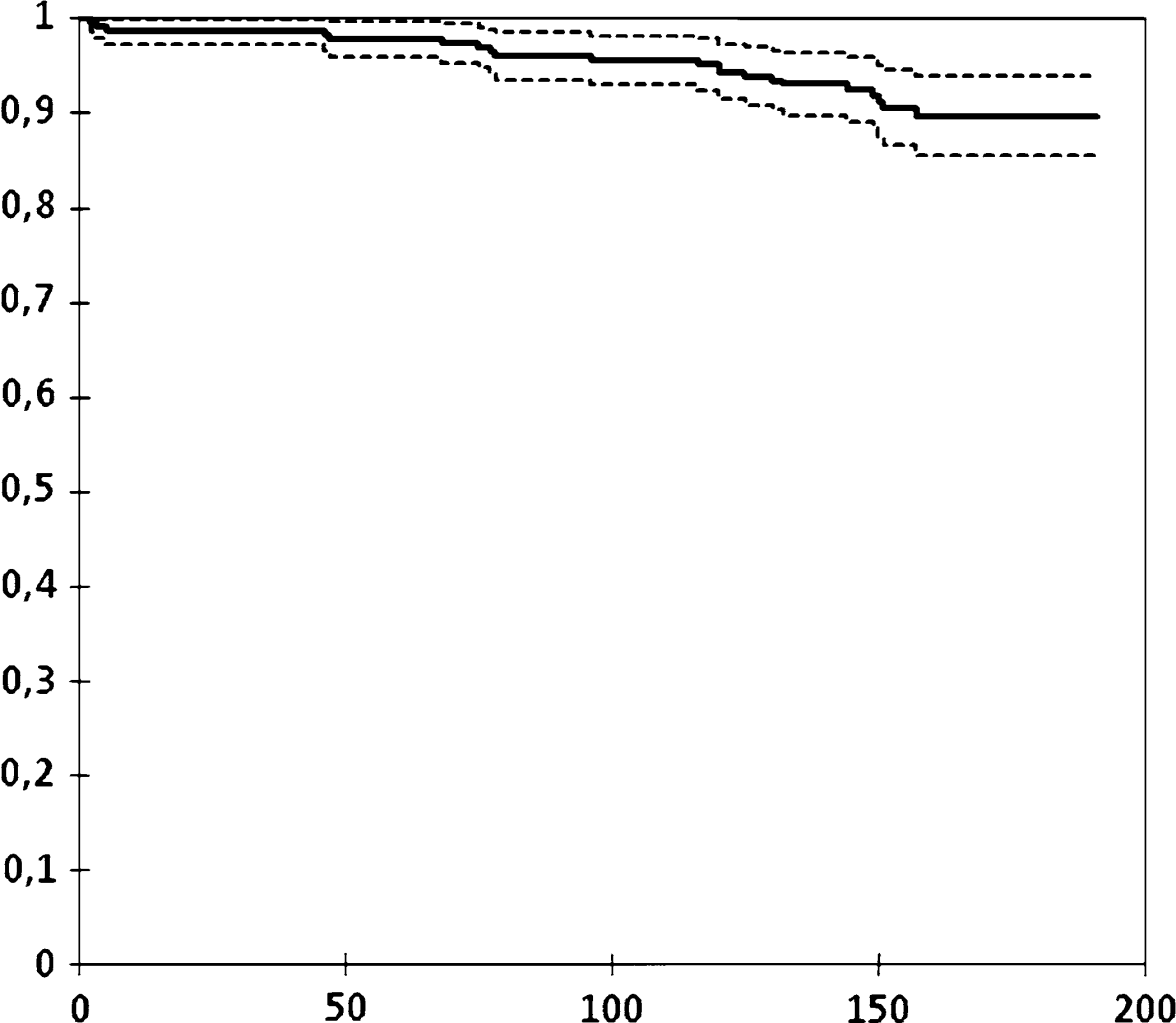
Kaplan–Meier curve of survival rate with failure with implant revision as the endpoint. Time reported in months

**Fig. 2:**
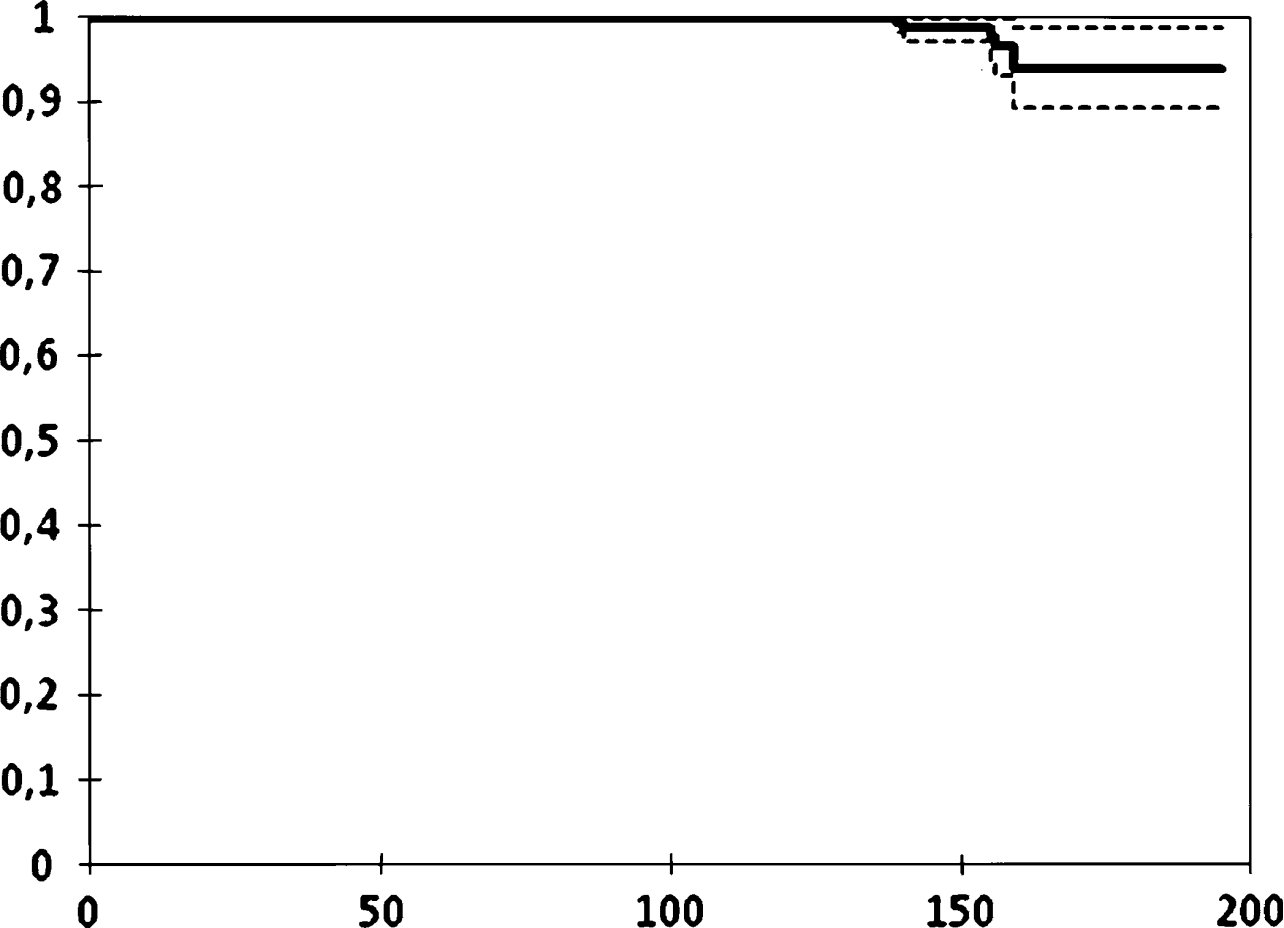
Kaplan–Meier curve of survival rate with Hospital for Special Surgery (HSS) total knee score of ≤60 as the endpoint (limit between satisfied and dissatisfied patients). Time reported in months

**Fig. 3:**
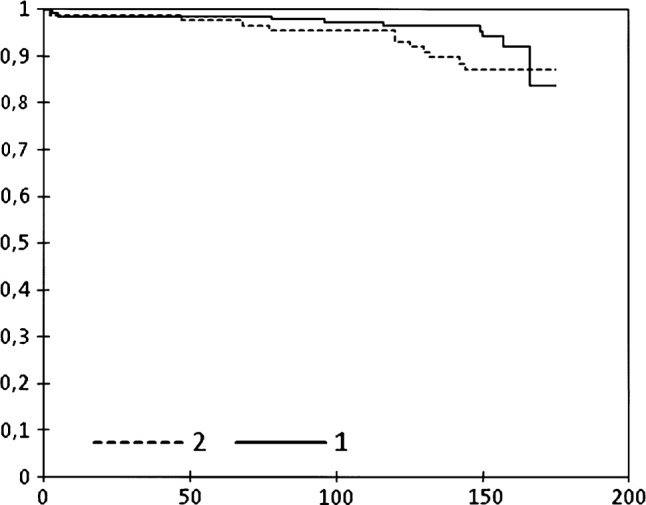
Comparison between resurfaced patella (group 1, *continuous line*) and unresurfaced patella (group 2, *dotted line*) total knee arthroplasties. Kaplan–Meier curve of survival rate with failure with revision as the endpoint. Time reported in months

**Fig. 4:**
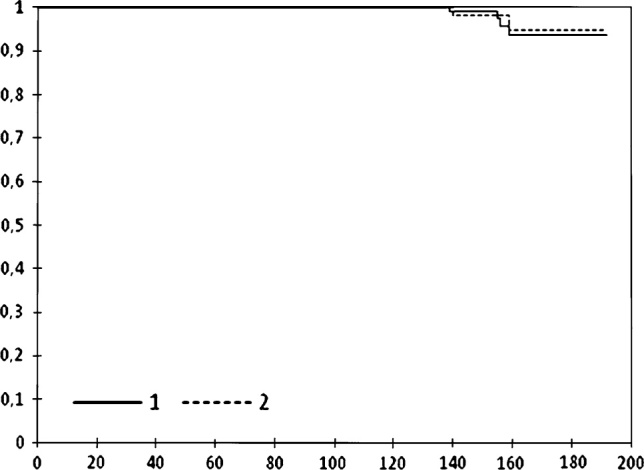
Comparison between resurfaced patella (group 1, *continuous line*) and unresurfaced patella (group 2, *dotted line*) total knee arthroplasties. Kaplan–Meier curve of survival rate with Hospital for Special Surgery (HSS) total knee score of 60 points as the endpoint. Time reported in months

Prosthesis failure requiring revision surgery was necessary in 20 cases (11.1%): 16 aseptic loosenings, two septic loosenings, one ligamentous severe instability and one supracondylic fracture. Cumulative average implant survival rate at 15 years was calculated according to the Kaplan–Meier method. Taking failure with knee arthroplasty revision as the endpoint, the cumulative average survival rate at 15 years was 90.6% ± 2% SD (Fig. 1). Taking an HSS score <60 points (corresponding to the limit between satisfied patients and unsatisfied patients) as the endpoint, the cumulative aerage survival rate at 15 years was 94.1% ± 2.4% SD (Fig. 2). The two different groups of patella substitution and conservation were compared for revision and patient satisfaction. The cumulative average survivorship at 15 years was calculated: taking implant revision for any reason as the endpoint, it was 81.3% ± 6.6% SD for TKA with patellar replacement and 83.8% ± 8.4% SD for TKA without patella replacement, even if the resurfaced patellar knee showed better survival rate at midterm (Fig. 3). Using a total score of ≤60 points as the endpoint, it was 93.6% ± 7.4% SD and 94.7% ± 8.7% SD, respectively (Fig. 4). In all these cases, there was no statistically relevant difference in terms of survival and patient satisfaction between the two methods (respectively, *P* = 0.916 and *P* = 0.210). Rates of failure and reintervention on the patella for patellar problems were analysed in both groups. No revision surgery was performed for patellar reasons in the resurfaced patella group. Conversely, in the group with unresurfaced patella, revision surgery for patellar causes was performed in four cases: one lateral release, one patellar replacement for persistent pain and two of extensor apparatus realignment.

## Discussion

This study represents a nonselected sample of PFC TKA with a fixed bearing and is based on a clinical and radiographic evaluation. Its strength is the large number of patients, the long follow-up and the independency of the study. Clinical results were good, with a significant increase in HSS total and partial scores. Also, patients’ range of motion and ability improved significantly, thus confirming the validity of the technique and the PFC implant. Cumulative average implant survival rate at 15 years, taking TKA revision for any cause as the endpoint, was approximately 90%, as reported in the literature [13–16]. Cumulative average survival rate at 15 years was calculated using HSS score of ≤60 points or less as the endpoint, which can be considered as the division between satisfied and dissatisfied patients. Such an analysis has rarely been reported in the literature but is meaningful because it provides the real degree of patient satisfaction [10]. In this case, the cumulative percentage of patients satisfied with the implant at 15 years was approximately 95%. The two values are different because some patients with radiographic signs of osteolysis and mobilisation have no pain. In these cases, revision surgery was performed before a dramatic failure in order to conserve bone stock and ligamentous stability.

Several aspects were studied to identify variability of practical value for predicting the durability of TKA implants. First, the early postoperative complications were evaluated, as the total knee replacement is a major surgery in which complications can occur: the incidence of such adverse events in this series is similar to that reported in literature [8, 9]. Moreover, the results suggest that when an accident (thrombosis, superficial early phlogosis and other minor complications) is treated immediately, there is no significant impact on implant survival.

Similarly, implant alignment was considered. We obtained a correct physiologic alignment along the frontal mechanical axis plus/minus approximately 3°, whereas in the lateral view, the femoral component had a flexion of approximately 3° to avoid femoral notching (potential cause of fractures). Angle values were similar to those reported in the literature [7, 17]. Then, comparison between alignment angles in the TKA group that underwent revision and those in the unrevised implant group showed no statistically significant differences, thus confirming, as recently reported in the literature, that, within a physiologic range, the postoperative mechanical axis does not affect implant survival rate [18].

In this study, the selective patellar replacement was adopted with good results. There were no statistically meaningful differences in terms of implant failure and clinical results between patients with a replaced patella and those without. Nevertheless, a secondary surgery on the patella was performed in the unresurfaced group. This, plus the fact that the patella was usually replaced in knees with the worse anatomic condition would suggest that patella replacement is preferable [7, 19]. No progressive radiolucent lines were found in approximately half of the patients but they are not signs of early loosening. In particular, there is evidence for a predominance of radiolucent lines in the vicinity of the femoral-stem apex (zone 1 femoral) and the medial tibial plateau (zone 1 tibial). Similar data were described by Rodricks et al. [13]. Conversely, results show that progressive radiolucent lines (14.3% in this study) correlate with implant failure in 100% of cases [14, 15].

In conclusion, this study shows that the PFC prosthesis with fixed bearing, cruciate sacrifice and cemented fixation is a good prosthetic solution. Early complications, mechanical axis in the physiologic range and patellar treatment do not correlate with implant failure. Progressive radiolucent lines, conversely, are predictive of a negative result independent of when they appear.
